# Modular Evolution of Coronavirus Genomes

**DOI:** 10.3390/v13071270

**Published:** 2021-06-29

**Authors:** Yulia Vakulenko, Andrei Deviatkin, Jan Felix Drexler, Alexander Lukashev

**Affiliations:** 1 Martsinovsky Institute of Medical Parasitology, Tropical and Vector Borne Diseases, Sechenov First Moscow State Medical University, 119435 Moscow, Russia; vjulia94@gmail.com (Y.V.); felix.drexler@charite.de (J.F.D.); 2 Department of Virology, Faculty of Biology, Lomonosov Moscow State University, 119234 Moscow, Russia; 3 Laboratory of Molecular Biology and Biochemistry, Institute of Molecular Medicine, Sechenov First Moscow State Medical University, 119435 Moscow, Russia; andreideviatkin@gmail.com; 4 Institute of Virology, Charité-Universitätsmedizin Berlin, Corporate Member of Freie Universität Berlin and Humboldt-Universität zu Berlin, 10117 Berlin, Germany; 5 German Centre for Infection Research (DZIF), Associated Partner Site Charité, 10117 Berlin, Germany

**Keywords:** coronavirus, evolution, recombination, spike

## Abstract

The viral family *Coronaviridae* comprises four genera, termed *Alpha-, Beta-, Gamma*-, and *Deltacoronavirus.* Recombination events have been described in many coronaviruses infecting humans and other animals. However, formal analysis of the recombination patterns, both in terms of the involved genome regions and the extent of genetic divergence between partners, are scarce. Common methods of recombination detection based on phylogenetic incongruences (e.g., a phylogenetic compatibility matrix) may fail in cases where too many events diminish the phylogenetic signal. Thus, an approach comparing genetic distances in distinct genome regions (pairwise distance deviation matrix) was set up. In alpha, beta, and delta-coronaviruses, a low incidence of recombination between closely related viruses was evident in all genome regions, but it was more extensive between the spike gene and other genome regions. In contrast, avian gammacoronaviruses recombined extensively and exist as a global cloud of genes with poorly corresponding genetic distances in different parts of the genome. Spike, but not other structural proteins, was most commonly exchanged between coronaviruses. Recombination patterns differed between coronavirus genera and corresponded to the modular structure of the spike: recombination traces were more pronounced between spike domains (N-terminal and C-terminal parts of S1 and S2) than within domains. The variability of possible recombination events and their uneven distribution over the genome suggest that compatibility of genes, rather than mechanistic or ecological limitations, shapes recombination patterns in coronaviruses.

## 1. Introduction

Coronaviruses are ubiquitous viruses infecting mammals and birds. They belong to the family *Coronaviridae* and are divided into four genera, *Alphacoronavirus* (α-CoV), *Betacoronavirus* (β-CoV), *Gammacoronavirus* (γ-CoV), and *Deltacoronavirus* (δ-CoV). Coronaviruses (CoV) are an important source of emerging infections due to their capacity to switch hosts and establish infections in novel host species [1,2]. Members of the genera α-CoV and β-CoV exclusively infect mammals, while γ-CoV and δ-CoV infect mainly birds, although they have also been found in cetaceans and pigs. Phylogenetic studies suggest that bats are the major source for α-CoV and β-CoV infection of other mammals [3,4]. Analysis of bat coronaviruses in China showed that α-CoV change hosts more frequently than β-CoV [5], and host-switching occurs between more distantly related bats. However, β-CoV is more relevant as a source of threatening novel human viruses, including SARS-CoV-2 [6,7,8]. Wild birds are the reservoir hosts for highly diversified γ- and δ-CoV [9].

The CoV genome is a large (26–32 Kb) single-stranded RNA of positive polarity, and includes one long open reading frame (ORF) of about 20 Kb that encodes (via a frameshift) two polyproteins, pp1a and pp1ab, followed by four ORFs that encode essential conserved proteins termed S(pike), E(nvelope), M(atrix), and N(ucleocapsid), as well as up to eight ORFs that encode accessory proteins [10]. The spike protein, which is the major receptor-binding protein, consists of two major domains, the apical S1 domain that carries receptor binding sites, and the basal S2 domain. S1 may be further divided into N-terminal and C-terminal domains (S1-NTD and S1-CTD). The accessory proteins are required for virus replication, but vary in number and are often non-homologous even among closely related viruses, hinting at past recombination events during their evolution [11].

Recombination is a general feature of RNA viruses [12] and a well-known feature of coronavirus genetics [13]. It was first described in murine hepatitis viruses (MHV) in 1985 [14]. Since then, isolated recombination events have been reported in most known coronaviruses. Multiple reports describe recombination between coronaviruses infecting both the same and distinct host species. Moreover, several events of recombination between coronaviruses and viruses from other families have been suggested [15,16,17]. All three novel zoonotic human CoVs (SARS, MERS, and SARS-2) have been shown to have recombination in their evolutionary history [6,7,8].In particular, spike domain exchanges are an important evolutionary mechanism reported in a number of coronaviruses and have been termed “modular evolution” of the spike protein [13,18,19,20,21,22,23,24,25,26]. Understanding the limitations of recombination at the level of coronavirus genera is essential for predicting their capacity to yield novel viruses. However, despite multiple isolated reports, there has been no comprehensive analysis comparing patterns among the four genera. Here we aim to systematically analyze recombination in four coronavirus genera.

## 2. Materials and Methods

### 2.1. Preparation of Nucleotide Sequence Alignments

All available complete genome sequences of viruses belonging to the genera *Alphacoronavirus* (n = 1260), *Betacoronavirus* (n = 17083), *Gammacoronavirus* (n = 452), and *Deltacoronavirus* (n =162) were downloaded from the GenBank database as of July 2020. Only the sequences of five essential genes (full ORF1ab, S, E, M, and N) present in all coronavirus genera were chosen for the analysis. The coordinates of ORF1a, ORF1b, and genes S, E, M, and N were extracted from GenBank annotations, and the nucleotide sequences of each gene were excised and aligned separately based on their corresponding amino acid translations using mafft [27]. The resulting alignments of distinct genes were concatenated. Entries with nucleotide sequence identity exceeding 99% were excluded from the alignment. Columns containing over 10% gaps were cut off using trimAl software [28]. The resulting alignments for α-CoV, β-CoV, γ-CoV, and δ-CoV consisted of 164, 122, 260, and 56 concatenated sequences, respectively (Table 1). Information on isolate, host, collection date, subgenus, and species was retrieved from GenBank entries and manually checked for all sequences in analyzed alignments (Supplementary Table S1). The Python scripts used for data processing are available at https://github.com/v-julia/coronavirus_recombination (accessed on 25 June 2021). The coordinates of spike protein domains were obtained from Swiss-Prot annotations [29].

### 2.2. Phylogenetic Analysis

The trees of ORF1a, ORF1b, and S nucleotide sequences were inferred using IQ-TREE with 10,000 pseudo-replicates, incorporating the best-fit model of nucleotide substitution, and rooted by midpoint for each genus [30]. The automatic coloring of taxa labels in ORF1a, ORF1b, and S trees was performed using a Python script (https://github.com/v-julia/coronavirus_recombination, accessed on 25 June 2021). Trees were visualized with FigTree v1.4.4 [31].

### 2.3. Recombination Analysis

**Figure 1:**
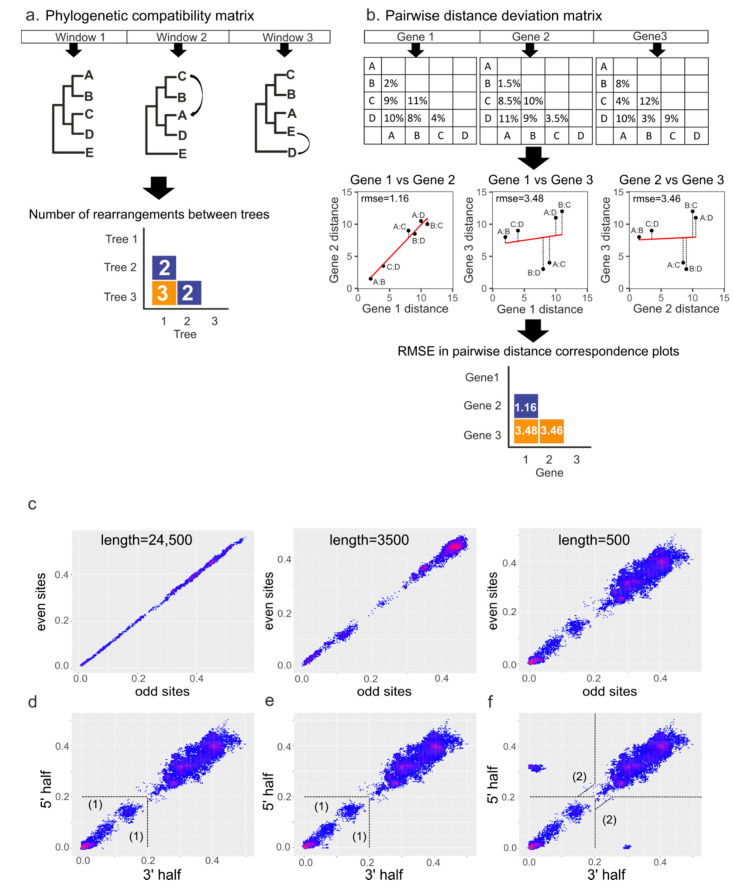
Principles of recombination detection methods. (**a**) illustration of compatibility matrix building algorithm [32,33]. For each window in the alignment, a phylogenetic tree is built, then the number of differences between obtained trees is calculated and stored in the matrix. The numbers of differences are usually normalized and visualized as a heatmap. (**b**) the algorithm for building pairwise distance correspondence plots (PDCP) and pairwise distance deviation (PDD) matrices. Pairwise genetic distances are calculated for genomic regions of interest. Then, each pair of sequence distances in genome region 1 and genome region 2 are plotted along the x and y axes, respectively, producing the PDCP. The overall divergence of all pairwise distances from the regression line is then estimated as the root-mean-square error (RMSE). RMSEs in plots for different genomic regions are visualized as a heatmap, making a PDD matrix. Simulated datasets were used to show typical PDCPs for alignments of different lengths without recombination (**c**), with one recombinant sequence in a 500-nt alignment (**d**), several recombination events between close clades (**e**), and recombination events between distant clades (**f**). Dotted lines indicate plot areas corresponding to recombination events between viruses differing by less than 20% nucleotide sequence in both genome regions (1), which were termed “recent”, and recombination events between viruses related by less than 20% in one genome region (2), termed “recent long-distance” for the purpose of discussion.

Two approaches were used for recombination analysis. The preliminary analysis of recombination patterns in separate genera was performed by computation of phylogenetic compatibility matrices [32,33] implemented in RDP4 software [34]. This method infers phylogenetic trees for different genomic regions using a sliding window and calculates the phylogenetic incongruence (Robinson-Foulds distance) between them (Figure 1a). The sliding window size was 600 nucleotides (nt) and the step size was 100 nt. 

The pairwise distance correspondence plot (PDCP), the second approach used for recombination detection and visualization, is based on testing whether the substitutions in different parts of the genome accumulated proportionally [35,36]. In this approach, distance matrices are built for two genomic regions and then plotted. Each point on the plot represents distances between a pair of sequences in these two genomic regions (pairwise genetic distance). Without recombination, the pairwise genetic distances between sequence pairs in two different regions should be well correlated and follow a linear relationship (Figure 1b, gene 1 and gene 2). The slope of the regression line will depend on the evolutionary rates in the two genetic regions. In the case of recombination between two regions, the pairwise distances belonging to a recombinant region will significantly diverge from the regression line (Figure 1b, gene 1 vs. gene 3, gene 2 vs. gene 3). If there have been many recombination events, multiple dots will deviate from the trend line. The extent of recombination between two genome regions can be expressed as the root-mean-square error (RMSE) of all pairwise distances from the regression line. The RMSE reflects the incongruences between genomic regions, and a higher RMSE may indicate relatively more recombination events involving the two genome regions analyzed. To illustrate recombination patterns across the genome in a set of virus sequences, distance matrices can be built for all possible genomic region pairs using a sliding window, and the RMSE for each possible pair of these windows can be visualized as a heatmap (Figure 1b, pairwise distance deviation matrix).

The approach described above was implemented as the R package “recDplot” (https://github.com/v-julia/recDplot, accessed on 25 June 2021) and online Shiny web application (https://v-julia.shinyapps.io/recdplot_app/, accessed on 25 June 2021). Both implementations utilize the alignment of virus sequences in the fasta format. PDC plots can display identifiers of virus pairs (according to sequence names in alignment) corresponding to distinct points. Information about virus species and host retrieved from GenBank (see *Preparation of nucleotide sequence alignments*) was used to interpret the results of PDC plots.

### 2.4. Simulation of Recombination Events

To illustrate how PDC plots work, alignments of nucleotide sequences containing one recombinant between closely related sequences (~5% nt distance), several recombination events between close clades belonging to the same viral species, and a gene transfer that could occur between different coronavirus subgenera (~30% nt distance) were simulated. Alignments of the genus *Alphacoronavirus* were used as a template. The alignment without recombinant sequences (“negative control”) was modeled by concatenating all odd positions and then all even positions of the template alignment. Thus, any effect of dissimilar mutation accumulation rates in distinct genome regions was negated, leaving only statistical noise (Figure 1c). “Negative control” alignments of 24,500, 3500, and 500 nt, which roughly corresponds to the length of the full genome, full *S* gene, and its receptor-binding domain, were generated to illustrate the effect of alignment length on noise (Figure 1c). Since the highest noise level was observed in the PDC plot of 500 nt length, this alignment was used to further simulate the recombination events. To create single recombination events, two sequences close on the phylogenetic tree were chosen arbitrarily, and half of the “recombination recipient” sequence was replaced by the corresponding sequence of the “donor” (Figure 1d). Several recombination events were modeled by choosing two close clades belonging to the same viral species on the phylogenetic tree and replacing half of the sequences from the recipient clade with the corresponding halves from the donor clade (Figure 1e). The same algorithm was applied when creating ancient recombination events, but more distant clades belonging to different viral species were chosen (Figure 1f).

## 3. Results

**Figure 2:**
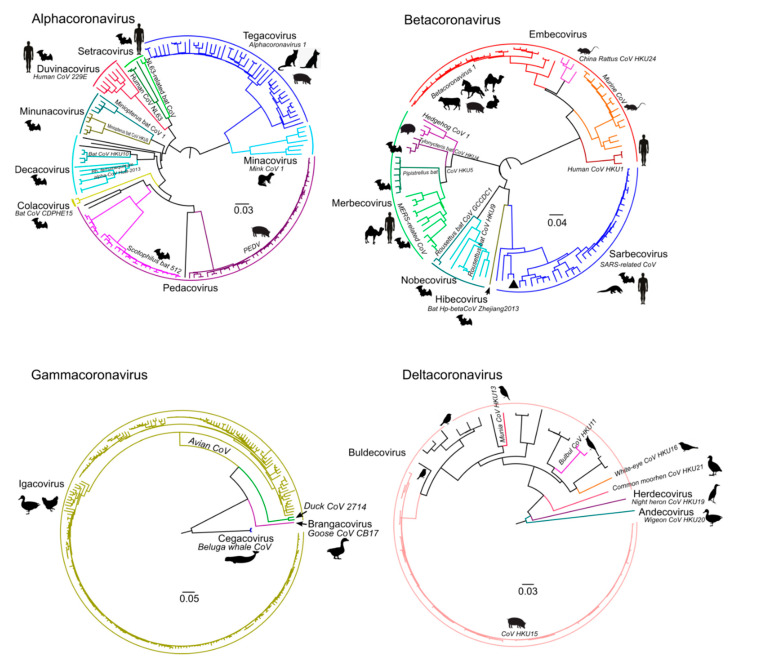
Datasets created for the four genera of coronaviruses represented as maximum likelihood phylogenetic tree of ORF1b, which contains RdRp. Subgenera names and species names are noted. The silhouettes of major hosts infected by virus species are shown near the tips. Taxonomy reflects the ICTV 2020 update. Not all genomes are formally assigned to species yet, and for some, the host data is lacking.

The number of available genomic sequences for distinct CoV genera was uneven (Table 1). Furthermore, many genomes, especially within the γ-CoV and δ-CoV, originated from few relevant hosts, such as humans, chickens, and pigs (Figure 2). Some species, especially within the genus α-CoV, were lacking from the dataset.

Common recombination analysis tools are aimed at the detection of distinct recombination events (e.g., various algorithms contained within the RDP package [34]) and preservation of conserved phylogenetic groups across the genome [phylogenetic compatibility matrices [32] and TreeOrder scans [37]]. Compatibility matrices (Figure 1a) were used for reference (see below), but they do not indicate the depth of recombination events (i.e., if they occurred recently among closely related viruses, or were found in ancestral sequences), only their relative abundance across the genome. TreeOrder shows recombination events based upon pre-defined sequence groups and is less suitable for hypothesis-free exploration. Additionally, these methods rely on phylogenetic grouping, which detects isolated recombination events, but may fail in the case of numerous recombination events within the compared fragments, as was observed below. Our key interest was the extent and patterns of recombination among coronaviruses across the genome and the structure of their gene pools. To avoid bias from multiple recombination events and the corresponding detection methods, raw pairwise sequence distances were plotted (see Section 2). In the absence of recombination, substitutions would accumulate in two genome regions proportionally (simulated examples in Figure 1c). Actual non-recombinant sequences usually display slightly higher deviation from the trend line due to distinct selection pressures in different genome regions and stochastic accumulation of mutations, but generally follow a linear dependence.

Even a single recombination event would result in the deviation of several points from the regression line because it would be reflected in many pairwise sequence comparisons between the recombinant sequence and its close relatives (Figure 1d). Multiple recent recombination events create a cloud-like distribution of points at close genetic distances (Figure 1e), while ancestral gene transfers are reflected in many descendant sequences and produce “cloud groups” (Figure 1f). The app (https://v-julia.shinyapps.io/recdplot_app/, accessed on 25 June 2021) allows the selection of individual points to identify potential recombinants quickly. Coordinates (genetic distances) of deviating points may suggest the timing of recombination events in the past. Substitution rates in CoV vary among virus taxa, virus hosts, and genome regions, between 10^−4^ and 6.0 × 10^−4^ substitutions per site per year (s/s/y) [38]. A rough average estimate of the substitution rate of 5 × 10^−4^ s/s/y implies the most recent common ancestor of coronaviruses that differ by 10^−2^ (1%) nucleotide sequence existed about a decade ago. Of course, this estimate should be used with care because distinct viruses might have different substitution rates [39], and at higher genetic distances saturation of substitutions and other mechanisms may lead to errors in molecular dating of several orders of magnitude [40].

Individual PDC plots indicate recombination between two genetic regions. The multitude of PDC plots for all possible genome region pairs can be further summarized into a pairwise distance deviation (PDD) matrix. In this approach, distance matrices are built for all genome regions using a sliding window, then for each possible pair of regions (a single PDC plot), the root-mean-square error (RMSE) of points from the regression line is calculated. Next, RMSEs from all possible pairs of genome regions are visualized as a heatmap (Figure 1b). A higher RMSE indicates lower overall congruence between two genome regions, which may be caused by recombination. The method is insensitive to the loss of phylogenetic signal and can detect frequent recombination that limits the sensitivity of many classical methods.

**Figure 3:**
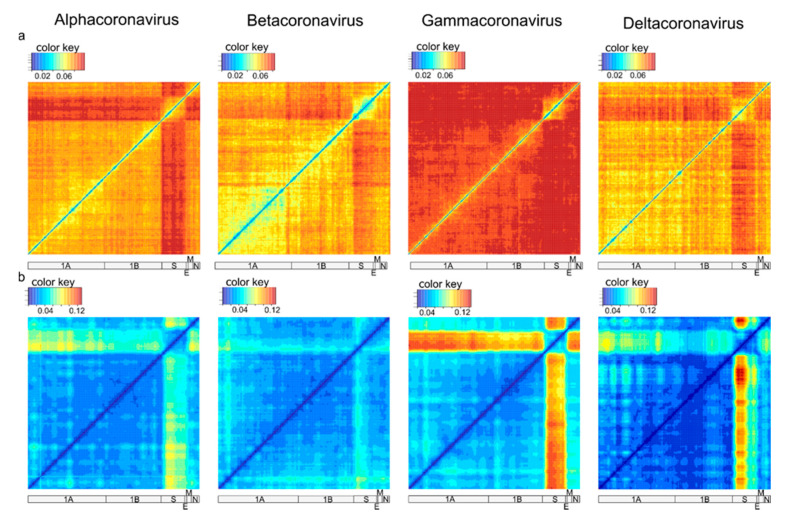
Recombination incidence in coronavirus genome detected by phylogenetic compatibility matrices based on normalized Robinson-Foulds distances (**a**) and pairwise distance deviation matrices (PDDM) based on deviations of points in PDCPs (**b**) for four Coronavirus genera. Spike gene is visually more involved in recombination relative to the rest of the genome in the four CoV genera. Colors reflect the values of normalized Robinson-Foulds distances and RMSE in PDCPs, respectively.

**Figure 4:**
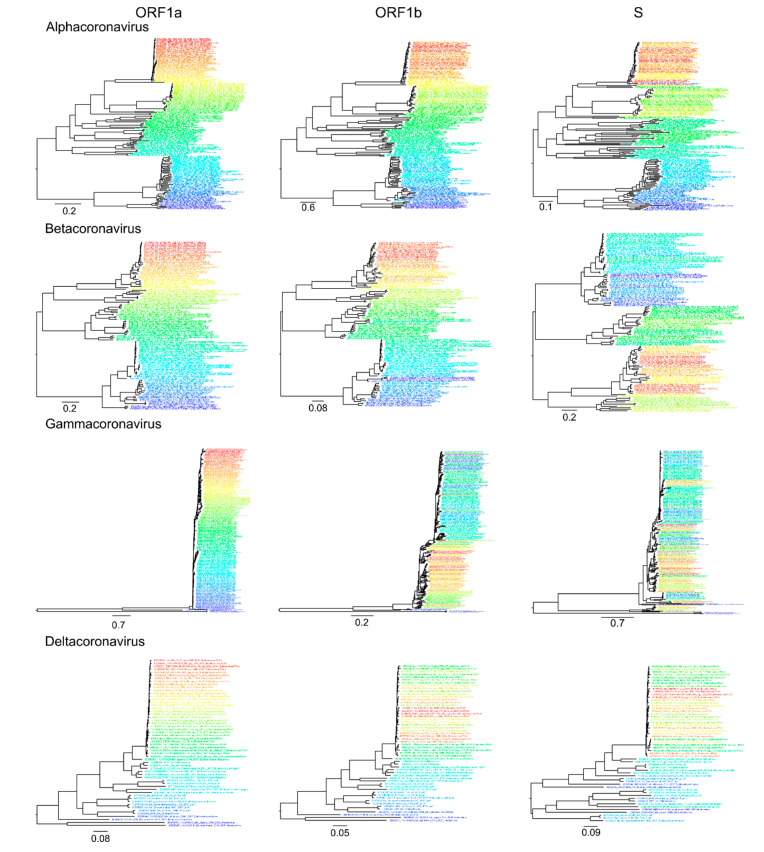
Taxon mixing in maximum-likelihood phylogenies of *ORF1a*, *ORF1b,* and *S* genes for four coronavirus genera. The colors of taxa names match in trees for each genus.

Analysis of recombination in coronaviruses was performed independently for α-CoV (n = 164), β-CoV (n = 122), γ-CoV (n = 260), and δ-CoV (n = 56). Preliminary tests suggested that a window size of 500–1000 nt allows reliable detection of recombination without losing resolution. First, a set of genome-scale recombination detection tools was used for a hypothesis-free recombination analysis. A traditional phylogenetic compatibility matrix (Figure 3a) showed that recombination occurred across the whole coronavirus genome. The spike gene was more often recombinant relative to the rest of the genome within the four CoV genera, as illustrated by approximately 1.5 times more phylogenetic conflicts in the phylogenetic compatibility matrix. Recombination prevalence was lower in the remaining parts of the genome in α-CoV, β-CoV, and δ-CoV, but almost evenly high across the genome in γ-CoV, as indicated by the higher number of phylogenetic conflicts between all genome regions in compatibility matrices (Figure 3a). We suggested that the overall prevalence of recombination along the genome in γ-CoV was so high that the compatibility matrix could not detect additional recombination involving the S gene. In order to check this, phylogenetic trees were built for ORF1a, ORF1b, and S using the same dataset (Figure 4). The ORF1 tree was colored by a gradient, and the same colors were used for each taxon on the other trees. Indeed, the degree of taxon mixing was visibly higher between γ-CoV ORF1a and S than between γ-CoV ORF1a and ORF1b; however, as only about half of the nodes were supported with robust bootstrap values, quantitative analysis was not done.

Recombination within the S gene was limited in all four species, but variations regarding the most preserved S fragments could be suggested. Thus, this classical method confirmed previously observed ubiquitous recombination between S and other genes [13,25] and suggested more common recombination in γ-CoV, but did not provide a detailed image of recombination events. 

The pairwise distance deviation matrices were generally consistent with phylogenetic compatibility matrices (Figure 3b). Recombination could be suggested between any pair of genome fragments; there were no genome regions completely devoid of recombination. The clearest results were obtained with a window size of 500 nt; other window sizes can be explored online at https://v-julia.shinyapps.io/recdplot_app/, accessed on 25 June 2021. There were two groups of genes that featured relatively less frequent recombination within the groups, but common recombination between them: (1) S and (2) all other genes except S. The boundaries of these groups were very clear in γ-CoV. In the other three genera (especially in β-CoV), the 5′ part of S had a greater degree of phylogenetic incompatibility relative to other genes than the 3′ part. Therefore, PDD matrices showed much lower compatibility of genetic distances between S and the rest of the genome, suggesting more common recombination, consistent with phylogenetic compatibility matrices. They also provided additional detail regarding the boundaries of recombining regions compared to phylogenetic compatibility plots. Then, individual PDC plots for genome regions suggested by PDD matrices were analyzed for detailed recombination patterns. 

**Figure 5:**
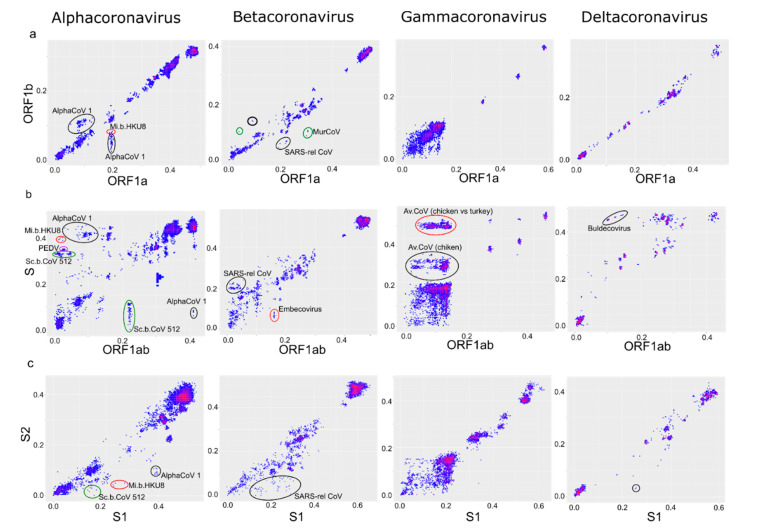
Recombination is more common between ORF1 and spike than within ORF1. Correspondence of pairwise nucleotide distances (PDC plots) between ORF1a and ORF1b (**a**), ORF1ab and spike (**b**), and S1 and S2 regions of spike (**c**). AlphaCoV 1, *Alphacoronavirus 1*; Mi.b.HKU8, *Miniopterus bat coronavirus HKU 8*, Sc.b.CoV 512, *Scotophilus bat coronavirus 512*; SARS-rel. CoV, *SARS-related coronavirus*; MurCoV, *Murine coronavirus*; Av.CoV, *Avian coronavirus.* Points that correspond to virus pairs that underwent recombination and are discussed in the text are marked with circles.

PDD matrices (Figure 3b) suggested a moderate prevalence of recombination within ORF1 in all four genera, with some potential hot-spots in ORF1a. PDC plots of ORF1a vs. ORF1b confirmed that the recombination level was moderate in α-CoV and β-CoV (Figure 5a), as also seen in the phylogenetic trees (Figure 4). There was evidence of a number of recombination events within ORF1ab, for example, between isolates of the species *Alphacoronavirus 1* infecting different animal hosts (Figure 5a, black circles) and between members of *Miniopterus bat coronavirus HKU8* (Figure 5a, red circle). Additionally, there were several recombination events within the species *Murine coronavirus* (Figure 5a, green circles) and *SARS-related coronavirus* (Figure 5a, black circles), including recombination between SARS-CoV-2 and SARS-related coronaviruses from bats (bold black circle). However, in general, there was a fair correlation of genetic distances between ORF1a and ORF1b. Notably, even in the absence of distinct recombination events, actual pairwise distances were not always perfectly correlated (here and below) as in the model data (Figure 1c). This is compatible with the ubiquitous moderate recombination suggested by PDD matrices (Figure 3b), phylogenetic trees (Figure 4), and with PDC plots using smaller genome regions that suggested additional phylogenetic incompatibility (not shown, may be explored via online tool at https://v-julia.shinyapps.io/recdplot_app/, accessed on 25 June 2021). In γ-CoV, there was a cloud of genetic distance pairs below approximately 10%, suggesting a frequently and promiscuously recombining gene pool among closely related viruses (Figure 5), again compatible with the thorough mixing of related taxa between, for example, ORF1a and ORF1b, observed on phylogenetic trees (Figure 4). δ-CoV showed no recombination within ORF1ab, likely because recombination events suggested by PDD matrices involved only very similar viruses, as seen in phylogenetic trees (Figure 4), and were not distinguishable from noise in the PDD matrices. Concordant with the phylogenetic trees, in all genera, PDD matrices did not indicate recombination within ORF1 between very distantly related viruses within a genus, and all recombination events were suggested within species.

PDD matrices (Figure 3b) suggested small-size recombination hot-spots within ORF1 in all CoV genera. A detailed analysis of these genome regions confirmed a higher incidence of recombination (Figure S1). Importantly, these hot spots were different in the four genera and could be located only using PDD matrices, not phylogenetic compatibility matrices (Figure 3a). Within some of these recombinant regions, there were very high maximum genetic distances (up to 70%). Manual inspection of the alignment at these regions was poorly compatible with the homology of the most divergent fragments. These findings could be theoretically explained by acquisitions of fragments from non-coronavirus ancestors; however, BLAST search failed to reveal a putative acquisition source.

ORF1 codes for a non-structural polyprotein, while S, E, M, and N are structural proteins. Nevertheless, only the S gene stood out from the other CoV ORFs as a frequently recombining gene. PDD matrices did not suggest notably more recombination between ORF1, E, M, and N genes than within ORF1 (Figure 3b), and PDC plots between the ORF1, E, M, and N genes were in principle similar to plots between ORF1a and ORF1b (Figure S2). Thus, in terms of recombination patterns, there was no clear distinction between ORF1 and the “minor” structural genes, and the latter were not analyzed further.

The most prominent pairwise distance disparity suggesting frequent recombination was observed between S and other genome regions (Figure 3). However, PDD matrices do not distinguish few major (ancestral) recombination events from many recent events, because both may cause deviation of many points from the regression line. To further investigate genus-level recombination patterns, individual PDC plots for genome regions suggested by PDD matrices were used (Figure 5b). A common feature of all CoV was a poor correlation of genetic distances between ORF1ab and S involving moderately related viruses (below 20–25% genetic distance). In these boundaries approximately corresponding to subgenera, there were many distinct recombination events in all CoV genera. In α-CoV, the examples include recombination between viruses of *Alphacoronavirus 1* species infecting different animal hosts (dogs, cats, pigs) (Figure 5b, black circles), and recombination within *Scotophilus bat coronavirus 512* species (Figure 5b, green circles). In β-CoV, recombination was seen within SARS-related coronaviruses and within the subgenus *Embecovirus* that includes the *Betacoronavirus 1* species (Figure 4b, black circle and red circle, respectively) In γ-CoV, prominent recombination was seen between members of the species *Avian Coronavirus* and *Avian Coronavirus 9203* isolated from chicken and ducks, respectively. An online tool available at https://v-julia.shinyapps.io/recdplot_app/ (accessed on 25 June 2021) allows further exploration of these figures for individual partners that correspond to the outlier points. Some of these recombination events were very recent because the viruses diverged by just a few percent of the nucleotide sequence in one of the compared genome regions. Many more recombination events could be suspected, and at low genetic distances, there was effectively a “cloud of genes” within species evident as a loss of correlation between genetic distances. In the case of γ-CoV, all virus pairs in the cloud of genetic distances belonged to the species *Avian Coronavirus* and *Avian Coronavirus 9203* that together comprise all turkey coronaviruses, all the genotypes of infectious bronchitis virus (IBV), and some duck coronaviruses. The vast majority of γ-CoV full genomes were generated from chicken IBV due to the high veterinary importance of this virus. *Avian Coronavirus* members isolated from other hosts were underrepresented in our dataset (Figure 2, Supplementary Table S1). Thus, the presence of many chicken IBV and just one duck coronavirus sequence in genetic distance clouds could be due to sampling biases instead of biological reasons. In δ-CoV, such a “cloud of genes” could be neither confirmed nor rejected due to the low number of available sequences and overrepresentation of closely related porcine viruses. 

Relatively recent recombination between more distantly related viruses (over ~25% nucleotide sequence distance in one genome region) was evident in α-CoV, γ-CoV, and δ-CoV, but not in β-CoV. The most prominent example (Figure 5b, circled) are bat coronaviruses of the *Scotophilus bat coronavirus 512, PEDV*, and *Alphacoronavirus 1* (feline coronavirus, canine coronavirus, transmissible gastroenteritis virus) species belonging to the *Alphacoronavirus* genus. Another notable case is the cloud of recombination events between chicken and turkey *Avian coronavirus* in the genus *Gammacoronavirus*; and recombination between sparrow coronavirus HKU17 and the other unclassified members of the subgenus *Buldecovirus* in δ-CoV described previously [19].

To get a better view of the recombination patterns within the spike protein gene, we first plotted pairwise distances between the two major domains of spike, S1 and S2 (Figure 5c). Recombination within spike was more pronounced than within ORF1 (Figure 5a) but less common than between ORF1 and S (Figure 5b). Thus, spike was more often (but not always) exchanged as a whole. Recombination patterns within spike differed between the CoV genera.

Within the S gene, γ-CoV had a perfect “cloud” of numerous recombination events between the S1 and S2 domains at genetic distances below 20–25% (Figure 5c). This “cloud” was similar to that observed within γ-CoV ORF1ab, but with higher genetic distances typical to the S gene. There was evidence of multiple recent recombination events between rather distant partners (distances <2% in one half of S and >15% in another). In α-CoV and β-CoV, the “cloud” of genetic variants produced by recombination between S1 and S2 was less pronounced (suggesting less prevalent recombination than in γ-CoV) but more extensive in terms of genetic distance between recombinants, and markedly asymmetric. Very divergent S1 (up to 40% sequence distance in α-CoV and 30% in β-CoV) could be combined with closely related (<10% distance) S2, but not vice versa. In β-CoV, the majority of such recombination events were between SARS-related coronaviruses isolated from bats, humans, and pangolins (Figure 5c).

The number of known δ-CoV sequences is significantly lower than in the other three genera. Moreover, there are almost no δ-CoV with pairwise sequences in S or its domains in the range between 5–20%, where most recombination events were observed in other CoV genera. There were few obvious recombination events within the δ-CoV spike, but the number and diversity of known δ-CoV sequences preclude detailed analysis of recombination patterns and matching them to other CoV genera. For the same reason, the analysis of recombination within spike in δ-CoV was not very informative (Figure S4), and further analysis was done only for the three other genera.

**Figure 6:**
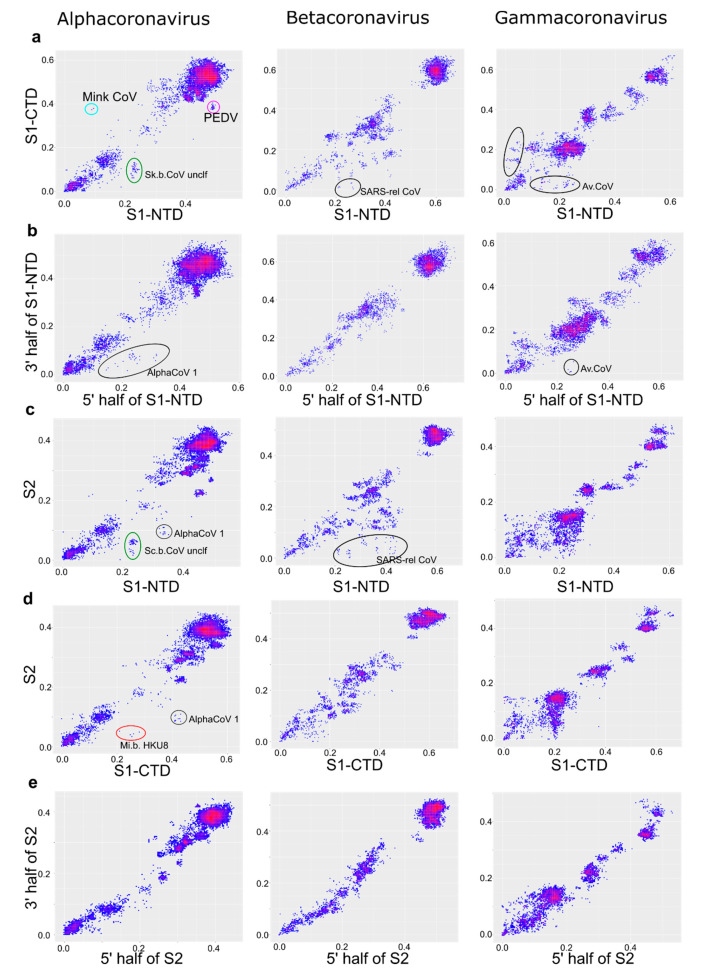
Recombination within domains of spike is less pronounced than between them. Correspondence of pairwise nucleotide distances in different regions of the spike protein: S1-NTD and S1-CTD domains (**a**), two halves of S1-NTD domain (**b**), S1-NTD domain and S2 region (**c**), S1-CTD and S2 region (**d**), two halves of S2 region (**e**). AlphaCoV 1, *Alphacoronavirus 1*; Mi.b.HKU8, *Miniopterus bat coronavirus HKU 8*, Sc.b.CoV 512, *Scotophilus bat coronavirus 512*; SARS-rel. CoV, *SARS-related coronavirus*; Av.CoV, *Avian coronavirus.* Points that correspond to virus pairs that underwent recombination and are discussed in the text are marked with circles.

The N-terminal part of spike (termed S1) comprises two major domains, the N-terminal domain (S1-NTD) and C-terminal domain (S1-CTD), both of which may contain receptor-binding sites. The shorter size of NTD and CTD fragments preclude quantitatively comparing recombination incidence to the S1:S2 pair, thus only overall patterns were noted. The recombination profile between S1-NTD and S1-CTD (Figure 6a) was generally similar to that between S1 and S2 (Figure 5c). There was very common recombination in γ-CoV at genetic distances below 25%, including many very recent transfers. Recombination between S1-CTD and S1-NTD in β-CoV included a number of long-distance events involving viruses that differed by up to 40% nucleotide sequence in S1-NTD. Similar to the pattern between S1 and S2 and S1-NTD:S2, recombination in β-CoV was asymmetric and included more divergent NTD than CTD or S2. Thus, most recombination events detected in β-CoV in the S1:S2 comparison (Figure 5c) corresponded to transfers of S1-NTD (Figure 6c). All recombination events observed in β-CoV were between SARS-related coronaviruses. Few relatively recent recombination events were seen between α-CoV S1-NTD and S1-CTD within *Scotophilus bat coronavirus 512* and *Mink coronavirus 1* species (Figure 6a, circles). PDC plots for S1-NTD:S2 and S1-CTD:S2 were similar to those for S1:S2, but enabled to resolve the approximate range of recombination events, which occurred only at the species level and involved viruses from *Alphacoronavirus 1*, *Miniopterus bat coronavirus HKU 8, PEDV*, and *Scotophilus bat coronavirus 512* species (Figure 6c,d).

There were notably fewer distinct recombination events within the spike domains (S1-CTD, S1-NTD, and S2) than between them. A generally more chaotic distribution of points could be largely affected by stochastic variation in a shorter genome region, as suggested by model data (Figure 1c). There were several recent recombination events within S2 between γ-CoV that were almost identical in one part of the domain and differed by up to 10% in the other (Figure 6e). A similarly low level or absence of recombination was seen within S1-CTD (Figure S3). The lack of recombination within domains was most pronounced in S2, especially in β-CoV, in stark contrast with recombination patterns between S1:S2, S1-CTD:S1-NTD, S1-CTD:S2, and S1-NTD:S2 (Figure 6). 

**Figure 7:**
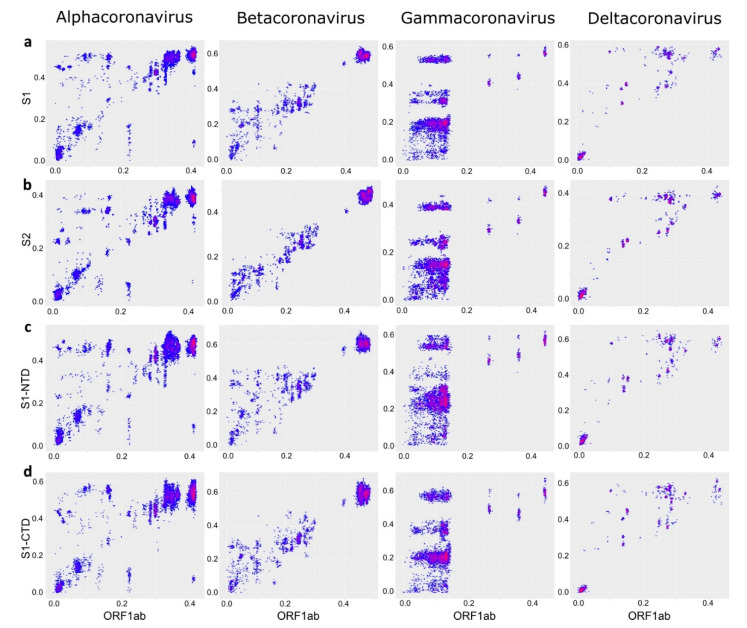
S1-NTD is the most mobile part of spike relative to the rest of the genome in β-CoV, while in the other genera spike is exchanged as a whole. Correspondence of pairwise nucleotide distances between ORF1ab and different regions of the spike protein: S1 (**a**), S2 (**b**), S1-NTD (**c**), S1-CTD (**d**).

PDD matrices suggest a distinct recombination profile in the β-CoV spike (Figure 3). Distinct recombination patterns were observed among CoV genera between different domains of spike (Figure 6). However, comparing just spike regions could be misleading regarding the direction of such transfers. Correspondence of pairwise nucleotide distances between ORF1ab and different regions of the spike protein confirmed that S1-NTD was indeed the most mobile part of spike relative to the rest of the genome in β-CoV, as indicated by more deviating pairwise distances and higher deviations, especially compared to the ORF1ab:S2 plot (Figure 7). In other genera, patterns were very similar when comparing ORF1ab to distinct domains of spike, confirming that spike is commonly exchanged as a whole.

## 4. Discussion

Recombination requires co-infection of an organism and a cell (limitation one), and its patterns depend on both mechanistic concerns (limitation two–formation of a recombinant genome and compatibility of genome fragments) and fixation of recombinants in a virus population (limitation three–selective advantage, frequency, and narrowness of bottlenecks). 

This study found distinct recombination patterns in mammalian and bird coronaviruses, and in different genome regions. No single theoretical limitation named above could explain all recombination profiles. It is known that recombination within a cell is very frequent; in MHV the recombination frequency per passage has been estimated to be 25% [41]. Experimental mechanistic studies in MHV suggested that recombination is almost random across the genome within a relatively short time frame, but after serial passages of recombinant viruses in tissue culture, particular cross-over regions were preferred, most likely as a result of selection pressure [42]. Later studies have shown that the recombination frequency increases progressively from the 5′ to 3′ end of the genome, probably corresponding to the transcriptional activity of the subgenomic negative strands [43]. Therefore, no specific mechanistic recombination constraints are known, and distinct recombination patterns observed in natural settings are more likely the result of ecological limitations and selection of viable recombinants. 

Compatibility of genome fragments is likely to be the reason for limited recombination within ORF1 (or, precisely, survival of recombinants) between distantly related viruses. On the contrary, recombination between significantly less similar viruses (with nucleotide distances up to 40%) was observed between ORF1ab and S, especially in α-CoV and γ-CoV. Some of these recombination events have been described previously [19,23,44,45,46], and this study confirms that this is a systematic pattern. Interestingly, the E, M, and N genes behaved similarly to the ORF1ab and differently from the spike, despite their location in different parts of the genome. This observation further stresses that compatibility rather than mechanistic and ecological possibility strongly affect the apparent (visible) recombination patterns. These compatibility barriers seemingly do not act at genetic distances below 3–10% in α-CoV and β-CoV, and 10–25% in γ-CoV, because below this limit recombination was ubiquitous in most settings (even within otherwise conserved spike domains), and closely related CoVs exist effectively as a cloud of genes in all genome regions. In this study, the gene cloud is best exemplified by γ-CoV and was less obvious in other genera. This is because, at low genetic distances, it is not distinguishable from statistical noise. Ubiquitous recombination generating a gene cloud in α-CoV and β-CoV is evident from the published similarity plots and bootscan graphs that bear evidence of multiple gene transfer events [47,48,49]. 

Genetic boundaries of these ubiquitously recombining gene clouds among similar viruses could also be affected by co-infection possibility and frequency. Relatively divergent γ-CoVs that have the most extensive gene cloud in all genome regions compared to other genera were isolated predominantly from a sole host (chicken) (Figure 2, Supplementary Table S1). Livestock trafficking and immunization with live vaccines could further increase co-infection chance and recombination incidence [50] and complement the formation of this gene cloud. Known α-CoV and β-CoV were isolated from many host species. Recombination between viruses infecting different hosts would require crossing the species barrier, a hurdle absent in chicken γ-CoV. Indeed, many recombination events among α-CoV and β-CoV were seen here and reported previously between viruses infecting the same host [23,24,47,51,52,53]. There are also examples of recombination between viruses from distinct hosts, both in this analysis and in previous publications [7,19,21,26,44,46,54,55,56,57]. However, the analysis above indicates that the incidence of such events was apparently not sufficient to form a gene cloud, as seen in γ-CoV in all genome regions. Thus, host specificity (and thus co-infection probability) defines the “short-distance gene cloud” among CoVs.

Compatibility of the ORF1ab vs. spike was apparently not a critical limitation within any of the CoV genera. A lower observed range of ORF1ab-spike recombination among β-CoV (in terms of the spike divergence) may be an artifact resulting from different modular flexibility of the β-CoV spike, where S1-NTD, rather than the whole spike, was the commonly transferred block (see below). Thus, from the point of view of emerging coronaviruses, any existing spike (or part of it) can end up in any ORF1-E-M-N context. 

Divergent spikes were commonly observed in viruses with closely related ORF1 genes, but not vice versa. This is best exemplified by γ-CoV, where spikes of turkey and chicken CoV differed by more than 40%, but highly divergent ORF1 genes could not be found in viruses with similar S (reported by Jackwood et al. in 2010 [45]). It remains to be studied if the repertoire of spike genes in the biosphere is indeed greater than that of other CoV genes, or whether some CoVs with highly divergent ORF1 genes have not been sampled yet. The first suggestion would mean that spike and the rest of the genome have distinct evolutionary patterns within a genus: core genes (ORF1ab-E-M-N) stick together, while spikes provide a coating for the core genes and jump across viruses, including those infecting different species, more often and with fewer constraints, and thus can gain greater overall diversity in the biosphere. Such semi-independent evolution of genome regions, usually structural vs. non-structural, is common in other RNA viruses and might be a general pattern of virus existence [58].

Recombination patterns within the S protein also varied among coronaviruses. Chicken γ-CoV spikes comprise a homogeneous gene cloud with apparently no compatibility barriers between spike domains or their parts, including fragments of S1-NTD or S1-CTD. However, this pattern was observed here and reported previously [22,59] only between reasonably closely related viruses (less than 20–25% nucleotide sequence distance). Exchanges between more divergent chicken and turkey CoV involved only the whole spike [45], and there were no such long-distance recombination events within the spike. Similarly, in α-CoV, long-range recombination involved the full-size spike [23,24] (Figure 7), while recombination within spike between distantly related viruses was apparently less common and not systematic. In β-CoV, S1, or even S1-NTD, rather than the full spike, was the module more commonly transferred between distantly related CoVs (Figure 7) [26]. This is rather unexpected because, at least in SARS-related viruses, the receptor-binding domain is mapped to the S1-CTD [60,61]. This observation suggests that there is a compatibility constraint between S2 and ORF1ab in β-CoV, and recombination within spike rather than involving the full S (as in α-CoV and γ-CoV) was preferred. In all genera, there was much less genetic distance incongruence within the spike domains than between them. This illustrates very well the lower compatibility of domain “halves” than the whole domains, formulated previously as the concept of modular evolution [25]. Modular evolution of spike has been discussed previously, based on isolated recombination events. This analysis shows that it occurs systematically at a genus scale in all CoV genera and provides a tool to further explore recombination patterns in CoV and other viruses.

Unfortunately, neither classical methods nor the approach suggested here can be used to quantitatively measure recombination incidence. This could be done for narrow taxa with a genome region suitable for Bayesian phylogenetic analysis with a molecular clock [62], but is not feasible at a genus level, because it does not represent a holistic population, and molecular clock analysis may be wrong by orders of magnitude for such divergent sequences. Moreover, the available sample of sequences is biased towards viruses of social/economic significance (such as IBV), or viruses with known reservoir hosts (bats), thus we abstained from quantitative conclusions. 

Using the tools and data presented here, it is possible to assay the potential gene pool available to coronaviruses in different genome regions and also to infer unknown host switches involving viruses found in different hosts, because recombination requires co-infection. Recombination has been used previously as a host switch indicator to infer the origins of MERS-coronavirus [63], but it can now be extended to additional CoVs. PDD matrices allow visualization of recombination across a genome within a large virus group, independently of a phylogenetic signal. In the case of excessively frequent recombination, as observed in γ-CoV, this method was superior to phylogenetic compatibility matrices (Figure 3), although when recombination incidence was more modest, as in α-CoV and β-CoV, these approaches produced comparable results. PDD matrices could detect genus-level recombination hot-spots within ORF1 that were confirmed by PDCPs and predicted that, in β-CoV, S1-NTD, rather than the full S, is the preferable transferred module. Thus, distance-based methods may be a valuable addition to classical tools for genome-level recombination analysis. On the other hand, they purportedly sacrifice statistical support and have decreased sensitivity at lower genetic distances (below 5% in shorter genomic regions) compared to e.g., bootscans, and cannot be recommended as a sole recombination analysis method.

Another limitation of this study comes from sample bias. The number of known genomic sequences in distinct species may vary by order of magnitude (Figure 2, Table 1). Additionally, the known diversity of S genes is higher than of other CoV genes, and the data was lacking to give a definitive answer on the evolutionary implications of this, i.e., if there is a corresponding diversity of core genes in some currently unexplored reservoirs. This notion concurs with a failure to trace the precise origin of the SARS-CoV-2 virus. Thus, dozens of ecology studies done on coronaviruses after the SARS outbreak in 2002–2003 were apparently not sufficient to provide explicit coverage of the genetic diversity of CoVs.

## 5. Conclusions

Recombination in CoV is ubiquitous, and co-infection possibility, including species barriers, is the major factor in the recombination patterns at the genus level. This work, as well as previous mechanistic studies, suggests that when co-infection is possible, all possible recombination variants are quickly generated. Survival of such recombinants is limited by the compatibility of genome fragments, with patterns that are similar among CoV genera, but with important variations. These patterns could be considered for engineering chimeric coronaviruses for vaccine design and basic studies.

## Figures and Tables

**Table 1:** Datasets used for recombination analysis. Detailed sequence data provided in Table S1.

Genus	Number of Sequences in the Dataset	Length of the Alignment, nt
*Alphacoronavirus*	164	24,585
*Betacoronavirus*	122	24,690
*Gammacoronavirus*	260	24,870
*Deltacoronavirus*	56	23,840

## Data Availability

The data presented in this study are openly available in GitHub repositories https://github.com/v-julia/coronavirus_recombination, (accessed on 25 June 2021), https://github.com/v-julia/recDplot that do not issue DOIs (accessed on 25 June 2021).

## Supplementary Materials

The following are available online at https://www.mdpi.com/article/10.3390/v13071270/s1, Figure S1: “PDD matrices and PDC plots for regions with heterogeneous genetic distances within ORF1. Red circles indicate hot-spots on PDD matrices that were used to build PDC plots”; Figure S2: Correspondence of pairwise nucleotide distances in ORF1 and concatenated E, M and N genes”; Figure S3: “Correspondence of nucleotide distances in the two halves of the S1-CTD domain”, Figure S4: “Correspondence of pairwise nucleotide distances in different regions of the spike protein in deltacoronaviruses: S1-NTD and S1-CTD domains (a), two halves of S1-NTD domain (b), two halves of S1-CTD domain (c), S1-NTD domain and S2 region (d), S1-CTD and S2 region (e), two halves of S2 region (f)”; Table S1: “Datasets used for recombination analysis”.
