# Supplementary figures and images for: Modular Evolution of Coronavirus Genomes
Source: Viruses. 2021 Jun 29;13(7):1270. doi: 10.3390/v13071270 (PMC8310335; doi:10.3390/v13071270)

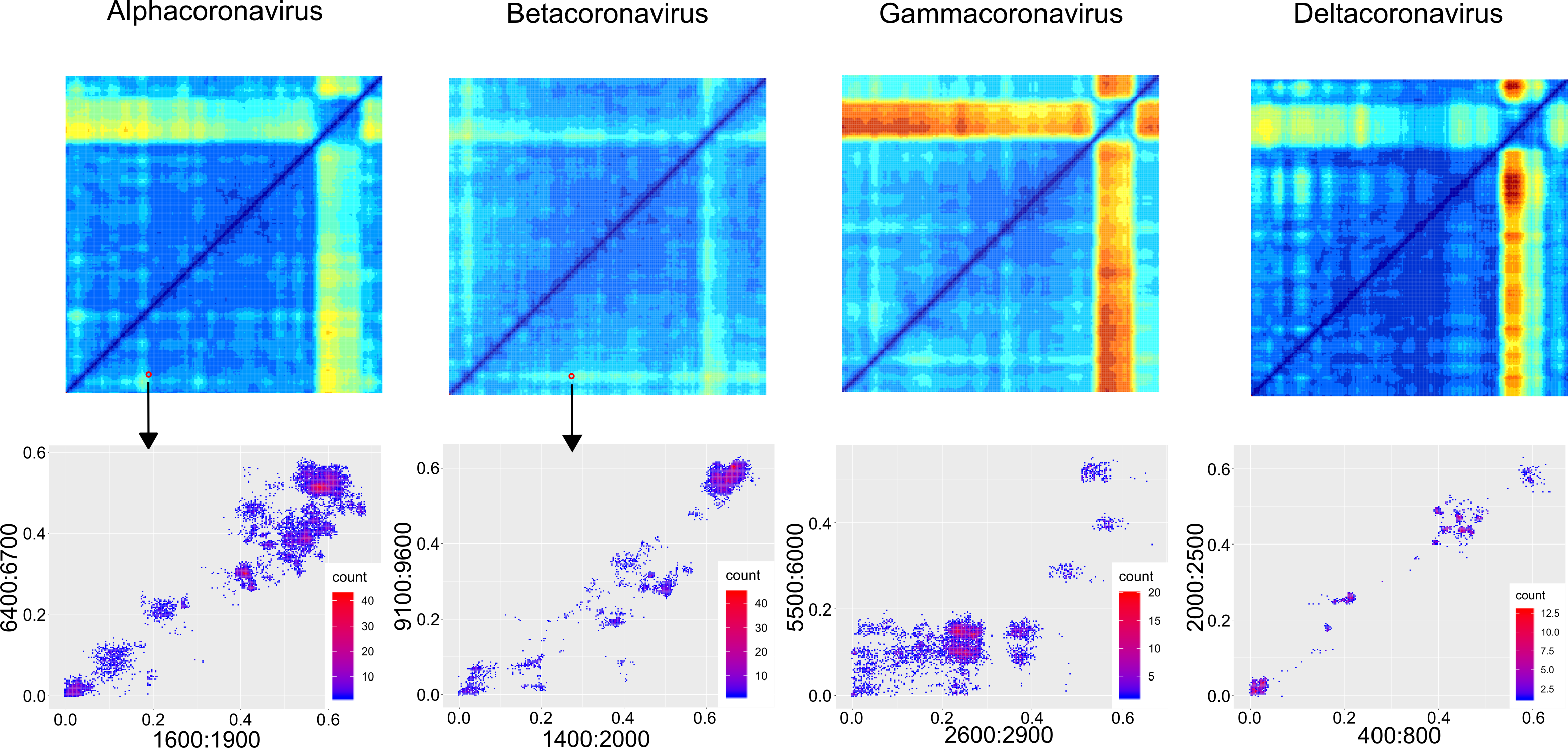

Supplement: Supplementary file 1 [file viruses-13-01270-s001.zip › FigureS1.png]

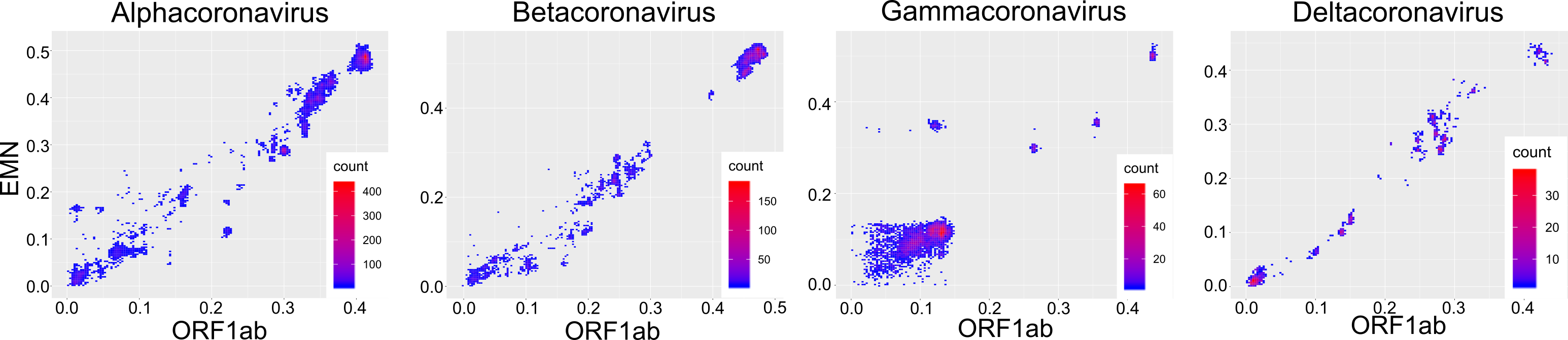

Supplement: Supplementary file 1 [file viruses-13-01270-s001.zip › FigureS2.png]

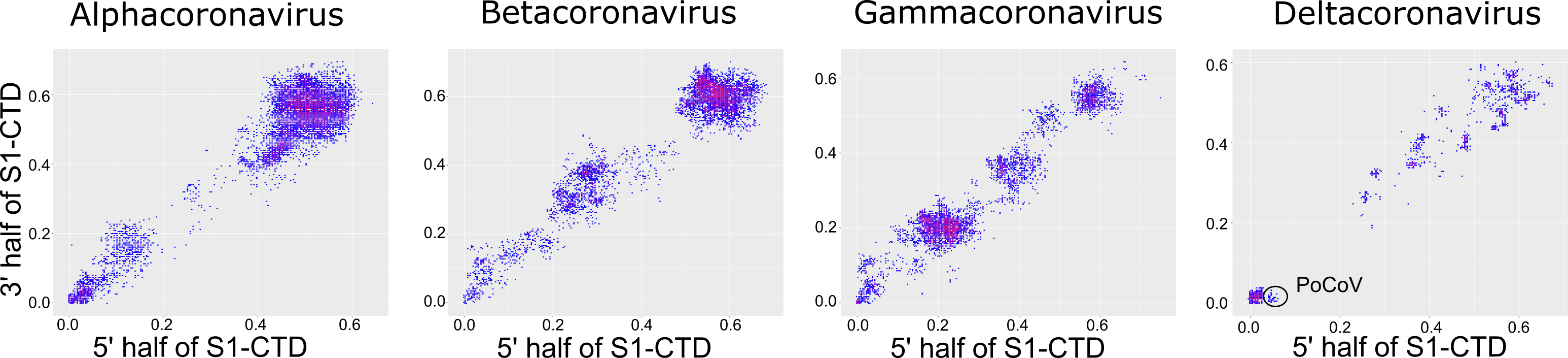

Supplement: Supplementary file 1 [file viruses-13-01270-s001.zip › FigureS3.png]

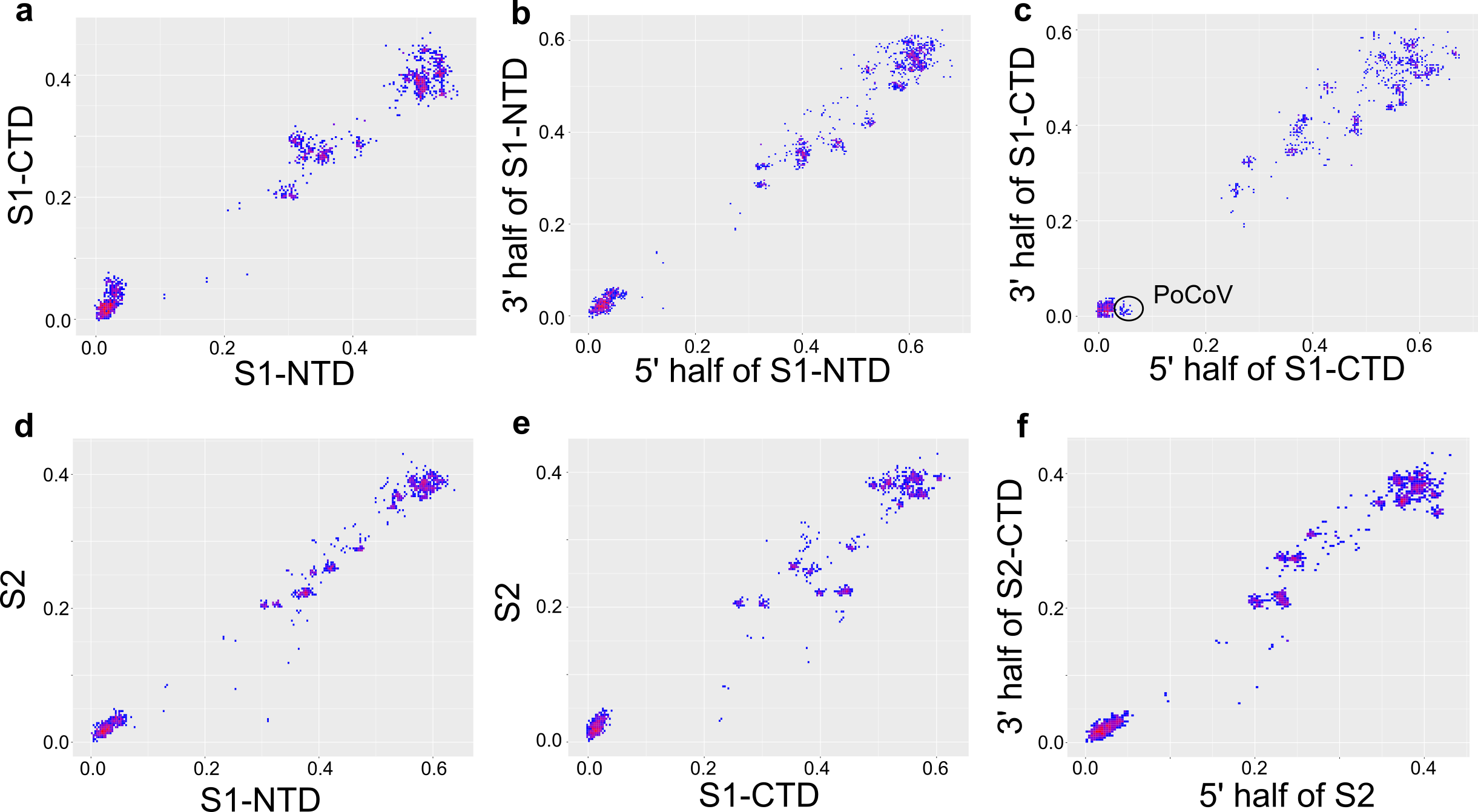

Supplement: Supplementary file 1 [file viruses-13-01270-s001.zip › FigureS4.png]
